# Mapping Determinants of Virus Neutralization and Viral Escape for Rational Design of a Hepatitis C Virus Vaccine

**DOI:** 10.3389/fimmu.2018.01194

**Published:** 2018-05-31

**Authors:** Mei-Le Keck, Florian Wrensch, Brian G. Pierce, Thomas F. Baumert, Steven K. H. Foung

**Affiliations:** ^1^Department of Pathology, Stanford University School of Medicine, Stanford, CA, United States; ^2^INSERM U1110, Institut de Recherche sur les Maladies Virales et Hépatiques, Strasbourg, France; ^3^Université de Strasbourg, Strasbourg, France; ^4^Institute for Bioscience and Biotechnology Research, University of Maryland, Rockville, MD, United States; ^5^Department of Cell Biology and Molecular Genetics, University of Maryland, College Park, MD, United States

**Keywords:** hepatitis C virus, vaccine design, epitopes, virus neutralization, antigenic domains, human monoclonal antibodies

## Abstract

Hepatitis C virus (HCV) continues to spread worldwide with an annual increase of 1.75 million new infections. The number of HCV cases in the U.S. is now greater than the number of HIV cases and is increasing in young adults because of the opioid epidemic sweeping the country. HCV-related liver disease is the leading indication of liver transplantation. An effective vaccine is of paramount importance to control and prevent HCV infection. While this vaccine will need to induce both cellular and humoral immunity, this review is focused on the required antibody responses. For highly variable viruses, such as HCV, isolation and characterization of monoclonal antibodies mediating broad virus neutralization are an important guide for vaccine design. The viral envelope glycoproteins, E1 and E2, are the main targets of these antibodies. Epitopes on the E2 protein have been studied more extensively than epitopes on E1, due to higher antibody targeting that reflects these epitopes having higher degrees of immunogenicity. E2 epitopes are overall organized in discrete clusters of overlapping epitopes that ranged from high conservation to high variability. Other epitopes on E1 and E1E2 also are targets of neutralizing antibodies. Taken together, these regions are important for vaccine design. Another element in vaccine design is based on information on how the virus escapes from broadly neutralizing antibodies. Escape mutations can occur within the epitopes that are involved in antibody binding and in regions that are not involved in their epitopes, but nonetheless reduce the efficiency of neutralizing antibodies. An understanding on the specificities of a protective B cell response, the molecular locations of these epitopes on E1, E2, and E1E2, and the mechanisms, which enable the virus to negatively modulate neutralizing antibody responses to these regions will provide the necessary guidance for vaccine design.

## Introduction

Estimation of worldwide prevalence of hepatitis C virus (HCV) infections ranged widely from 71 to 185 million people (1, 2) and roughly 400,000 will die annually from HCV associated liver failure and hepatocellular carcinoma (2). An estimated three million people are living with HCV infection in the United States, and there is an annual infection rate of 34,000 new infections (3). A contributing factor is the consequence of an opioid epidemic that shows no signs of slowing down and is unfortunately associated with increased injection drug use as a major mode to consume illicit opioids. When needles are shared by injection drug users, the risk of becoming infected with HCV increases. Of young adults (≤30 years) living in non-urban areas of states in the central Appalachia area infected with HCV, 73% had a history of injection drug use (4). Data from 2006 to 2012 in some states from these areas showed an astonishing 364% increase in infection amongst young adults. This was primarily in non-minority communities, with non-urban areas increasing from approximately 1.3–4.3 cases per 100,000 population while urban areas increased from 0.3 to 1.5 cases per 100,000 during this 6-year period (4). Because of this alarming rate of HCV infection and its potential lethality, the need for the development of a preventive vaccine is evident. It should be noted that effective direct acting antiviral drugs are available to treat HCV infections. However, susceptibility to reinfection after cure (5) and their prohibitive cost will limit their utility to contain this epidemic.

Vaccination is a powerful and proven method for infection prevention against many pathogens and a vaccine that is effective, accessible, and affordable is needed to control the further spread of HCV. This has been a difficult task because this virus is able to rapidly mutate and escape from protective immune responses. An understanding of what elements of a vaccine are needed and what challenges there are to guide vaccine design are discussed in this review. The focus is on the identification and functional characterization of conserved epitopes that elicit broadly neutralizing antibodies. We will review different immunogenic regions in the virus envelope E2 and in the covalently linked E1 and E2 heterodimer glycoproteins, and the challenges posed by regions of sequence diversity that contribute to viral escape from protective immunity. Collectively, the information gained will form the basis of rational structure-guided design of B cell epitopes in a reverse vaccinology approach to be included in the development of a preventative HCV vaccine (6–8).

## Traits of an Effective HCV Vaccine

While there is some debate whether B cell versus T cell responses are necessary for an effective HCV vaccine (9), a brief review of these responses during acute infection suggests that both arms of immunity will be required. During acute HCV infection, 20% of infected individuals will clear infection spontaneously while 80% develop a chronic infection. Spontaneous viral clearance has been associated with robust CD4+ and CD8+ responses that are maintained for several years after the virus has cleared. Losing the robust CD4+ response results in disease progression, hence the importance of the T cell response (10). Neutralizing antibodies elicited during acute infection also appear to contribute to spontaneous clearance. Early and strong neutralizing antibody responses were closely associated with HCV clearance (11, 12). Once viral clearance is achieved, neutralizing antibody levels either decrease or disappear. Those with absent or low serum neutralizing antibody levels during early infection and, therefore, with a delayed neutralizing antibody response pattern tend to develop chronic infections. Knowing that strong and early cellular and humoral immune responses to acute HCV infection is critical for spontaneous clearance, the implication is that both arms of a protective immune response are required to be induced in an effective vaccine design.

Experiences obtained from HIV vaccination studies indicate that antibody-dependent cytotoxicity (ADCC) could be an important determinant of protection (13). While it has been shown that certain subsets of natural killer (NK) cells are associated with disease progression and treatment outcome in chronic HCV patients (14), very little is known about the role of these effector cells during the acute phase of HCV infection. Both, NK cells and the highly abundant Kupffer cells are able to mediate ADCC. Recent publications suggest that ADCC mediated by non-neutralizing antibodies might be impaired in chronic HCV patients potentially due to increased cleavage of CD16 by host cell proteases (15, 16). However, the role of ADCC during acute HCV is only poorly studied. It remains to be determined whether ADCC contributes to viral clearance and might serve as an important determinant for antiviral protection. A key issue is the lack of clear evidence of infected cells expressing surface HCV-encoded antigens.

## Broadly Neutralizing Antibodies to HCV E2 Target Epitopes Containing Variable and Conserved Regions

**Figure 1:**
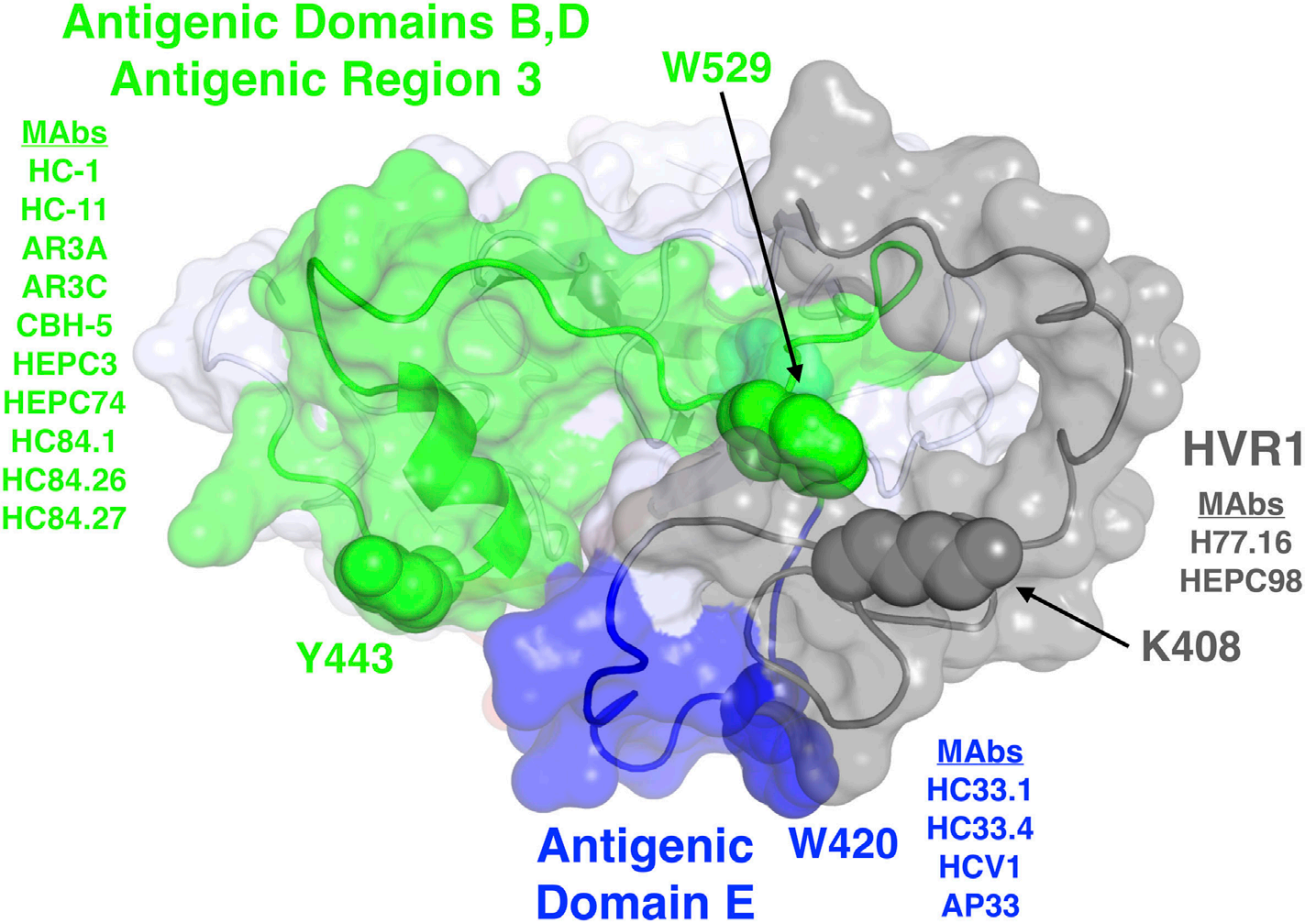
Structure and mapped epitope sites on the front layer, or neutralizing face, of E2. Structure shown is the E2 core crystal structure (23), with N-terminal residues corresponding to hypervariable region 1 (HVR1) and antigenic domain E (residues 384–423), which are mostly absent or unresolved from the E2 core crystal structure, modeled using the FloppyTail protocol in Rosetta (31) and shown for reference. Epitope sites are colored according to antigenic domains/regions or HVR1, with shared key epitope residues shown as spacefill and labeled. The remainder of E2 is shown as light gray. Representative monoclonal antibodies with binding residues mapped to each domain or HVR1 are also shown.

**Figure 2:**
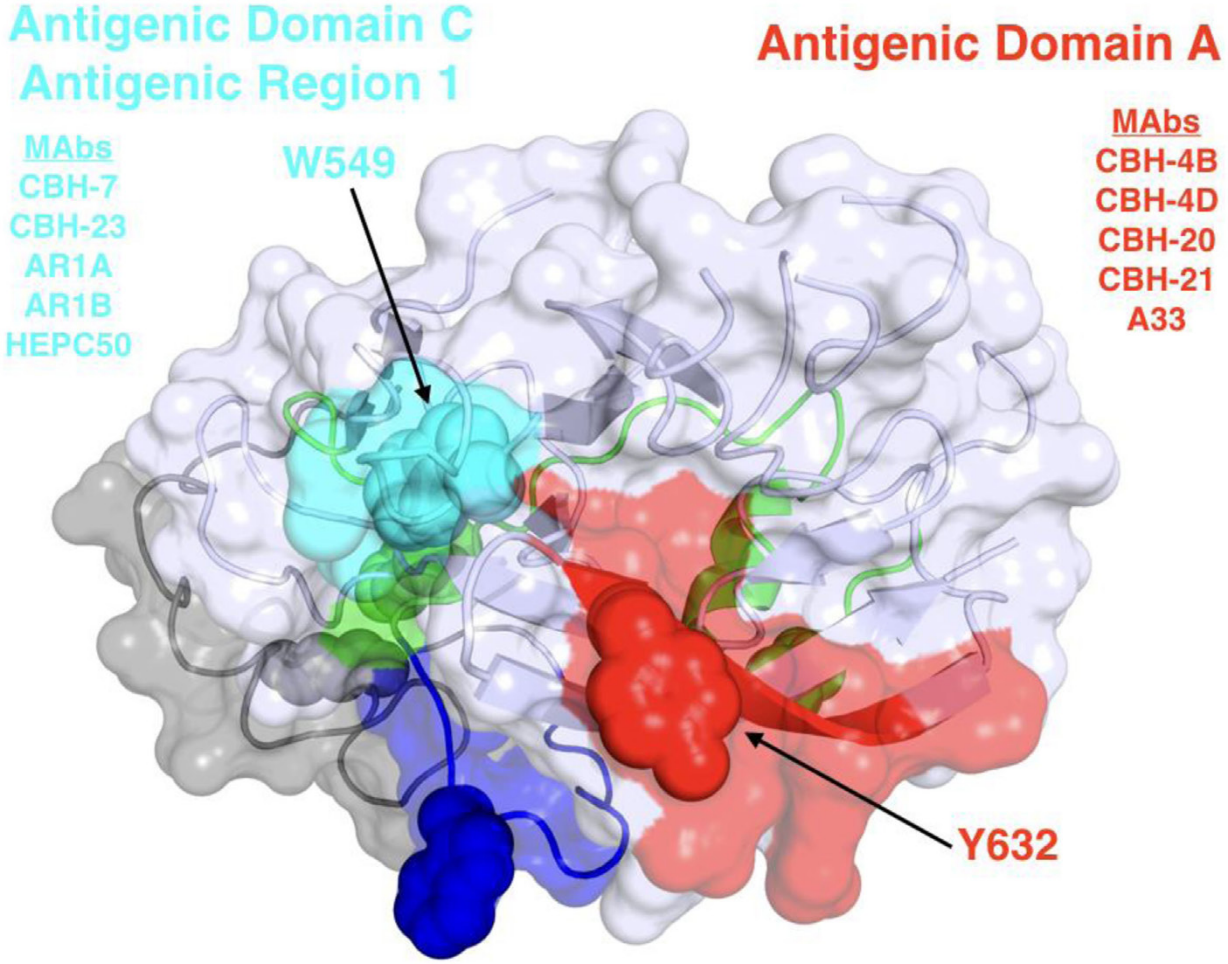
Structure and mapped epitope sites on the back layer and proximal β sandwich residues of E2. The structure corresponds to a distal face of the E2 core structure shown in Figure 1, with antigenic domains/regions labeled and representative monoclonal antibodies listed.

Broadly neutralizing monoclonal antibodies (MAbs) are an important guide for vaccine design for HCV and other highly variable viruses. These MAbs are directed mainly at epitopes in the E2 glycoprotein (17). The majority of human MAbs (HMAbs) isolated from infected individuals are to conformational epitopes in E2 and can be grouped in distinct clusters of overlapping epitopes. Two commonly employed nomenclatures are clusters designated as antigenic domains (18) or antigenic regions (AR) (19, 20). While there is substantial overlap between these two sets of overlapping epitope clusters, there are differences. The antigenic domains, A–E (Table 1) and AR1-3 (Table 2) are restricted to epitopes on E2. Two other ARs, AR4 and AR5, are clusters of epitopes requiring key residues on both E1 and E2 (Table 2). Generally, neutralizing MAbs, both human and mouse, are to epitopes on the exposed surface of E2 (Figure 1). The neutralizing HMAbs directed at epitopes within antigenic domains B, D, and E include key residues on E2 (residues W420, Y443, and W529 in Figure 1) that are also involved in virus binding to the HCV tetraspanin co-receptor, CD81, and thus mediate virus neutralization by blocking virus interaction with this required receptor for virus entry (21, 22). These epitopes are mostly conserved, which explains the wide breadth of virus neutralization of their associated antibodies. Antigenic domain B is also highly immunogenic and antibodies to this region are commonly found in the sera of HCV-infected individuals (21). Epitopes within domains B and D do overlap with shared contact residues in the 441–443 region [but antigenic domain D epitopes do not have residues 529–535 (Table 1)] and form a supersite of conformational epitopes on the exposed surface of the E2 core structure that contributes to CD81 binding (23, 24). Residues within antigenic domain B participating in virus binding to CD81 include 529, 530 and 535 (21). For antigenic domain D epitopes, the 441–443 region is involved in CD81 interaction (25). In the AR system, both antigenic domain B and D are included in AR3 (Figure 1; Table 2). Another highly conserved region is antigenic domain E located just downstream of hypervariable region 1 (HVR1) at a E2 segment encompassing residues 412–423 that also is responsible for broadly neutralizing antibodies (Table 1) (22, 26, 27). Antibodies to domain E are directed at mainly linear epitopes that include residue 420, which has been shown to be needed in virus binding to CD81 (28). A key residue that affects a broadly neutralizing HMAb, HC33.4 to domain E, is located at 408, within HVR1 (21, 22). Taken together, the ability to elicit antigenic domain B, D, and E antibodies will be important for vaccine design as these antibodies will prevent virus entry. Non-neutralizing antibodies are to overlapping epitopes on the back layer of E2 (Figure 2) and mainly in antigenic domain A (Table 1) and AR1 (Table 2). A shared residue for all domain A epitopes is 632. Interestingly, antigenic domain C, which mediates broad neutralization, is located in part at residues 544–549 in the central beta sandwich (residues 492–566), a region that is flanked by the front and back layers of the E2 core structure. Although some antigenic domain C residues are shared with AR1, domain C epitopes are exposed on the virus surface to allow access for antibody binding that leads to virus neutralization (Tables 1 and 2; Figure 2). This is further supported by the observation of overlap between the epitopes of CBH-7, a neutralizing antibody to domain C, and AR5A, a broadly neutralizing HMAb to E1E2 (19, 20). Global E2 and E1E2 antibody epitope mapping studies from Foung and Law lab groups have been described (29, 30).

**Table 1:** Immunogenic clusters as defined by antigenic domains.

Antigenic domain	Epitope location and key elements
Hypervariable region 1	384–410, mainly isolate-specific linear epitopes mediating neutralization with some interacting with SR-B1

E	412–423, mainly linear epitopes mediating broad neutralization

D	420–428, 441–443, 616, conformational epitopes on surface layer mediating broad neutralization with a residue 616 located in the back layer

B	431–439, 529–535, conformational epitopes mediating broad neutralization located on the surface layer

C	Conformational epitopes mediating broad neutralization located in part at residues 544–549 that is in the central beta sandwich (aa 492–566)

A	581–584, 627–633 conformational epitopes located on the back layer and mediating non-neutralizing antibodies

**Table 2:** Immunogenic clusters as defined by antigenic regions.

Antigenic region	Epitope location and key elements
1	E2 non-neutralizing face involving residues 495, 519, 544, 545, 547, 548, 549, and 632

2	E2 back layer region involving residues 625 and 628

3	E2 neutralizing face involving residues 427–443, 529–530. Residues 459, 499, 503, 558, and 616 influence folding of front layer, and residues 424, 425, 517, 518, 520, 523, 535, 536 influence folding of CD81-binding loop

4	E1E2 interface with specific residue 698

5	E1E2 interface with specific residue 639 and 665

Hepatitis C virus has multiple variable regions in E2 that aid the virus in evading from protective immunity. They include the HVR1 located at the N-terminus encompassing residues 384–410, hypervariable region 2, residues 460–485 and the intergenotypic variable region, residues 570–580. The HVR1 downmodulates protective immunity by at least three mechanisms. Although antibodies to this region are neutralizing, rapid mutation occurs leading to escape variants without compromised viral fitness that stay one step ahead of the antibody response (32). Another mechanism is shown with infectious recombinant cell cultured virions, HCVcc, with and without HVR1. Broadly neutralizing HMAbs neutralized HCVcc without HVR1 with greater potencies than against wild-type HCVcc (33, 34). Thus, it can be argued that a HVR1-deleted vaccine antigen could help boost broadly neutralizing antibody responses. *In vitro* studies with infectious HCV variants also help to define the region of HVR1 that is important for virus interaction with another HCV co-receptor, scavenger receptor class B type 1, SR-B1, and the region responsible for rapidly escaping variants to neutralizing antibodies (35).

Less is known about the immunogenic regions on E1 and E1E2. While a few HMAbs to E1 have been described (36, 37), two have been shown to exhibit broad virus neutralization (36). Two regions, AR4 and AR5, require contact residues on both E1 and E2, and some of these antibodies mediate broad virus neutralization (20). HMAbs to AR4 and AR5 do not mediate neutralization by blocking virus binding to CD81 but are postulated to inhibit conformational changes to the E1E2 heterodimer during viral entry. Because E1 has a relatively high degree of conservation within different genotypes and subtypes, elicited antibodies to E1 tend to exhibit broad neutralization. For these reasons, it is probable that vaccine constructs composed of both E2 and E1 in the form of heterodimers will be superior than vaccine constructs composed of E2.

## Viral Escape in the Defined Regions of the Epitope

The elusive virus is able to escape from the immune containment, in part due to its high rate of mutation and driven by presence of neutralizing antibodies. These mutations can occur either in areas directly targeted by neutralizing antibodies, or not directly targeted. CBH-2 is a neutralizing HMAb to an epitope within the antigenic domain B cluster. As discussed earlier, the epitope for CBH-2 has residues that participate in virus binding to CD81. However, there are at least two regions in E2 that are required to form the CBH-2 epitope. A conserved region located at 529–535 and a region at 425–431 with some variability. A single amino acid substitution at 431 results in viral escape from CBH-2 and a variant without compromised viral fitness (38). In contrast, another domain B antibody, HC-11 also requires these two regions to form the targeted epitope but the derivative escape variant with a mutation at 438 has reduced fitness. A third domain B HMAb, HC-1, is not associated with viral escape and its epitope has not been defined beyond the conserved region 529–535 (39).

Another region of great interest for vaccine development is the E2 segment 412–423 that is highly conserved. The importance of this region was first recognized by studies with a murine MAb, AP33 that recognizes mainly a linear epitope at 412–423 (40). However, a mutation at N417 that leads to a glycan shift to N415 will result in a robust variant able to escape neutralization by AP33 (41). A similar mechanism of escape has been documented with an HMAb designated as HCV1 (42). A different HMAb, HC33.1, to this region, interestingly, has a completely different neutralization profile. The variant with a N417 glycan shift is more sensitive to be neutralized than wild-type virus HC33.1 (41, 43). Structural studies revealed that this conserved E2 region, designated as antigenic domain E is highly flexible (43, 44). The implication for vaccine design is to stabilize domain E in a conformation that mirrors the HC33.1-bound epitope and not AP33 nor HCV1.

## Viral Escape Mutations Outside of the Defined Epitopes

Mutations that occur outside of epitope and receptor binding sites can also result in structural changes that will lead to escape from broadly neutralizing antibodies (45). These mutations lead to reduced efficiency of broadly neutralizing antibodies targeting different ARs, although viral fitness and efficiency of CD81 interaction are also reduced. Another example is studies with HC33.4 and AR4A, two neutralizing HMAbs to epitopes without any overlap and with different mechanisms of virus neutralization. The key binding region of HC33.4 is to the amino terminus of E2, 412–423, while for AR4A, it is the carboxy terminus of E2 along with residues on E1 to form its epitope (20, 22). HC33.4 is able to neutralize HCV by blocking interaction with CD81 and AR4A mediates neutralization by presumably the inhibition of E1E2 conformational change associated with viral entry. Unexpectedly, both antibodies have similar neutralization profiles against a large panel of genotype 1 isolates and both antibodies poorly neutralize variants with mutations at 403, a residue not known to be involved in the non-overlapping epitopes of these two HMAbs (46). Thus, extra-epitopic mutations need to be considered as well when discussing mutations that lead to viral escape. Escape mutations are especially important for HCV infected individuals receiving a liver transplant. Once the patient receives a new liver, reinfection of the liver by HCV is common as the virus is still able to avoid the immune system. Indeed, several of the escape variants isolated from patients undergoing liver transplantation were found to be located outside the defined epitopes. Many of these variants, selected post-liver transplantation, contained mutations within the CD81 binding domains II and III (47).

## Viral Escape Mutations by Altered Receptor Dependency

Accumulating evidence suggests that HCV can escape from broadly neutralizing antibodies that target envelope receptor-binding sites by altering the dependency on viral cell entry factors CD81 and SR-B1. For HCV, this has been first demonstrated for a cell culture adapted JFH1 variant that was characterized by an increased affinity for CD81: a mutation of E2 G451 to R resulted in increased E2 binding affinity to CD81 and at the same time reduced the dependency on the entry factor SR-BI. The mutant virus showed highly increased sensitivity to neutralizing anti-envelope antibodies (48). A similar effect has been observed for L403F and L438V that modulate resistance to the HMAbs HC33.4 and AR4A by altering E2 binding to SR-B1, as discussed above (46). Furthermore, engineered mutations of the E2 protein revealed nine different polymorphisms that were associated with reduced SR-B1 dependency and increased resistance against a panel of neutralizing antibodies (49). This indicates that escape *via* changes in SR-B1 binding efficiency is a relevant motif for HCV escape variants. A different mechanism has been observed for E2 residues 447, 458, and 478 that have been identified in clinically relevant transplant escape variants as important determinants for susceptibility to neutralizing antibodies (50). Mutations F447L, S458G, and R478C conferred resistance against conformational HMAbs CBH-2, CBH-5, CBH-23, and HC1 by altering the interaction of E2 with cell surface CD81. Furthermore, increased entry efficiency might also contribute to viral escape of these clinically relevant patient variants. Thus, changes in receptor usage by the HCV envelope protein is an important mechanism for viral escape that needs to be addressed in prospective vaccine studies or immunotherapeutic approaches using anti-E2 antibodies.

To address this challenge, antibodies targeting different HCV entry factors have been proposed for immunoprevention (51–56). More specifically, antibodies targeting SR-B1, CLDN1, and CD81 have demonstrated broad antiviral effects against HCV infections and have been suggested for the prevention of liver graft infection complementary to treatment with direct-acting antivirals (57). Single treatment with antibodies or small molecule inhibitors targeting viral receptors can potentially result in escape mutations as it has been reported for the SR-BI antagonist ITX-5061 (58) or during a clinical trial of an anti-CD4 monoclonal antibody targeting HIV-1 infection (59). Due to the reduced rate of transmission and the increased barrier for escape mutations, targeting of multiple entry host factors likely prevent viral escape due to changes in receptor tropism. Interestingly, when neutralizing antibodies that inhibit virus interaction against different co-receptors are tested simultaneously (as discussed more extensively in the next section), there can be antagonism or synergy in their combined effect (60, 61). Taken together, simultaneous targeting of multiple factors can lead to synergistic inhibition of infection (62), further strengthening this approach.

## Attributes of HVR1 that should be Considered in Vaccine Design

There are functions of HVR1 (aa 384–410) located at the N-terminus end of E2 that argue against and for its retention in vaccine design. It is highly immunodominant and virtually all HCV infected individuals will have serum antibodies to HVR1. The high rate of mutations in this region helps the virus escape protective antibody responses to this region by first serving as an immunological decoy (63). Second, it physically shields the more conserved antigenic domain B and D regions in E2 (33, 64). HVR1 partially blocks broadly neutralizing domain B and D antibodies from binding to their respective epitopes; removal of HVR1 leads to a defective virus that is more susceptible to these antibodies. Third, we recently proposed a new mechanism in which HVR1 adversely affect the function of broadly neutralizing antibodies (65). When some antigenic domain E HMAbs, e.g., HC33.4, were more extensively mapped, a residue located in HVR1 at 408 was found to affect HC33.4 binding to E2. This raised the possibility that when an anti-HVR1 antibody is bound to E2, it can interfere with the binding of HC33.4 and other domain E HMAbs. This was found to be the case as postulated with H77.16, a murine neutralizing MAb to HVR1 (60). Surprisingly, the binding of H77.16 also inhibited the binding and neutralization of antigenic domain B and D HMAbs. Additional studies showed that this interference is by steric hindrance and collectively supported the hypothesis that an anti-HVR1 response can interfere with more protective neutralizing antibody responses. Based on these observations, vaccine design should be based on a HVR1-deleted E1E2 immunogen to increase the production of broadly neutralizing antibody responses.

However, HVR1 also facilitates viral entry cells by interacting with SR-B1 and thus allowing attachment and eventual entry of the host cell *via* CD81. Although the majority of anti-HVR1 antibodies are isolate-specific, some antibodies to this region have broad neutralization profiles. J6.36, H77.39, and H77.16 are murine MAbs that target regions within HVR1 (Figure 1) (60). H77.39 inhibited binding of HCV to CD81 and SR-B1. While both J6.36 and H77.16 block E2 attachment to SR-B1, J6.26 also reduces E2 binding to CD81 (60). Binding studies revealed that H77.39 and H77.16 are reactive to all six major HCV genotypes. Therefore, antibodies to HVR1 could play a key role in vaccine design for their abilities to have a wide breadth of neutralization and for their ability to block SR-B1 attachment. This perspective has been supported by a recent study that shows a similar antibody to H77.16, HMAb HEPC98 (Figure 1), synergistically neutralize HCVcc when combined with a HMAb, HEPC74 that maps to an antigenic domain B region (61). Their observation contrasts with the observation that H77.16 when combined with other antigenic domain B or D antibodies led to antagonism (65). These contrasting results are likely the result of greater spatial separation between E2-bound HEPC98/HEPC74 relative to E2-bound H77.16/HC-11.

## Conclusion

This review outlines the functional organization of antigenic domains and epitopes within each domain that are associated with escape or are more invariant and essential for viral fitness, receptor binding, and viral entry. The findings raise the possibility of antagonistic relationship between immunogenic decoys, e.g., HVR1, which elicit antibodies associated with escape and may be responsible for antibody-mediated interference in the protective antibody response. Collectively, these studies begin to create a high-resolution map of conserved neutralizing epitopes not associated with viral escape and how other antigenic domains serve as diversions of the immune response and are able to elicit antibodies that negatively modulate neutralizing antibodies. Having this information, a future direction is to employ the knowledge in vaccine design. One approach is to stabilize flexible regions on E2 known to encode broadly neutralizing epitopes to be more favorable to antibodies that are not associated with rapid escape. For example, antibodies to the E2 region comprising residues 412–423 have broad neutralizing activities. However, an adaptive mutation in this linear epitope, Asn417Ser, is associated with a glycosylation shift from Asn417 to Asn415 that enables HCV to escape neutralization by MAbs such as HCV1 and AP33. By contrast, the HMAb HC33.1 can neutralize virus bearing the Asn417Ser adaptive mutation. Structural studies showed that E2_412–423_ when bound by this antibody has a distinct structure than either AP33 or HCV1 (44). The results highlight the structural flexibility of the E2_412–423_ epitope, which may serve as an evasion mechanism to reduce antigenicity. It is probable that other E2 regions with similar structural flexibility impede the induction of neutralizing antibodies by a similar mechanism.

## Author Contributions

All authors listed have made a substantial, direct, and intellectual contribution to the work and approved it for publication.

## Conflict of Interest Statement

The authors declare that the research was conducted in the absence of any commercial or financial relationships that could be construed as a potential conflict of interest.
